# A cross-sectional study of antimicrobial use among self-medicating COVID-19 cases in Nyeri County, Kenya

**DOI:** 10.1186/s13756-022-01150-7

**Published:** 2022-08-30

**Authors:** George Kimathi, Jackline Kiarie, Lydiah Njarambah, Jorum Onditi, David Ojakaa

**Affiliations:** 1 Amref Health Africa (H.Q)., P.O. Box 27691-00506, Nairobi, Kenya; 2 Division of Disease Surveillance and Response (DDSR), Nyeri County, Kenya; 3 BRIM RESEARCH, Code 00508 Yaya Towers, P. O. Box 76100, Nairobi, Kenya

**Keywords:** Self-medication, Antimicrobial resistance, Survey, Nyeri County Kenya, COVID-19 symptoms

## Abstract

**Background:**

During the COVID-19 pandemic, Nyeri County in Kenya was among the regions reporting a high number of confirmed cases. This exemplified the increased need of addressing potential antimicrobial resistance (AMR) and self-medication during disease outbreaks. This study examined the extent of self-medication with antimicrobials among COVID-19 confirmed cases in the County.

**Methods:**

A cross-sectional survey using phone-based interviews was conducted in August 2021 among a sample of 280 out of 2317 confirmed COVID-19 cases in the County using a pre-coded questionnaire. Descriptive analyses of frequencies and causal logistic regression were conducted using STATA version 13.

**Results:**

A total of 193 (68.9%) of the respondents indicated developing COVID-19 related symptoms-mainly cough (41.5%), headache (38.3%), and fatigue (34.7%). Over one-fifth (23.4%) of the respondents had self-medicated with antibiotics, 60.6% of whom did so at the onset of symptoms before the confirmatory test, and 51.5% self-medicating more than once. Common antibiotics used were Azithromycin (40.0%) and Amoxycilline (23.3%), with a considerable 21.7% having difficulty remembering the name of the drugs. Only half (50.4%) of the respondents (128/254) were aware of regulations towards self-medication with antibiotics. Age was the only socio-demographic variable significantly related to reduced self-medication, with older persons less likely to self-medicate. On the other hand, developing COVID-19 symptoms, awareness of COVID regulations, and appreciation of the need for self-medication awareness were related to increased self-medication.

**Conclusion:**

Being older, developing COVID-19 symptoms, and appreciating self-medication awareness have influential effects on the use of antimicrobials. Public health interventions should be timely during infectious disease outbreaks to prevent undesirable health-seeking behavior such as irrational antimicrobial use. AMR policies should enhance awareness of the risks of self-medication and address barriers that deter people from timely access of health services during disease outbreaks. Further research should be conducted on the self-medication and AMR nexus, especially during health emergencies.

**Supplementary Information:**

The online version contains supplementary material available at 10.1186/s13756-022-01150-7.

## Background

Antimicrobial resistance (AMR) is a challenge in low-income countries, resulting from inappropriate and irrational use of antimicrobials, more commonly known as self-medication [1–3]. It threatens the effective treatment of infections and is a public health issue with national and global dimensions [2]. It is one of humanity's greatest threats, with drug-resistant infections causing at least 700,000 deaths globally each year [3] and upto 4.2 million deaths in Africa by the year 2050 [4]. Further, according to this study, AMR influencers go beyond hospital settings and oversight. There is therefore need for the one health concept (a health service delivery method involving multiple disciplines working locally, nationally, and globally to attain improved health for people, animals, and the environment). 

Without intervention, it is approximated that mortality may reach 10 million deaths annually by 2050. This situation is now aggravated in developing countries like Kenya due to weak health systems and poor health-seeking behavior, especially during the COVID-19 response. Antimicrobial resistance leads to treatment failures, prolongs hospital stays, worsens clinical outcomes, and makes surgical procedures and chemotherapy risky and unsafe, burdening the already weak health systems. In the broader developing world—India—a study was conducted on demand for COVID-19 medication without prescriptions among community pharmacies [5]. It concluded that while there was an increase in the demand for medication (Hydroxychloroquine, Azithromycin, Ivermectin, and Vitamin C) without prescription, there was no evidence of an increase in the sales of antimicrobials, an encouraging development.

Ismail and others [6] have examined the results, effects, and suggestions for lowering habitual self-medication use among Africans. They define self-medication as one’s own initiative or on the recommendation of someone else, to use drugs, herbs, or home remedies without doctor consultation. According to the study, self-medication has attained a critical stage in Africa; some of the population use drugs no matter how toxic the material so long as the unprofessional advice is seen as a solution to the individual’s health problem. In neighboring Tanzania to Kenya, a study conducted on the related theme of antibiotic use among consumers in a suburb of the capital city, Dar es Salaam sheds light on gaps in health education [7]. The study concludes that most consumers interviewed at outpatient pharmacies in sampled public and private hospitals and marketplaces) have poor knowledge on rational use of antibiotics. A moderate percentage possess good knowledge of conditions treatable with antibiotics; high level of education and owning health insurance are associated with good knowledge of rational use of antibiotics. 

In Kenya, self-medication with antibiotics has been associated with late diagnosis of several conditions, affecting the outcome of the treatments. According to the Ministry of Health in Kenya, 70% to 80% of cancer of the cervix cases are diagnosed in late stages [8] due to delay as patients self–medicate to get better. There is no record of the national prevalence rate for self-medication with antibiotics (SMA) in Kenya. However, research conducted in Kisumu City, Western Kenya, showed that 74% of patients had practiced self–medication with antibiotics before visiting a health unit [9]. Other studies have also shown that 70% of pharmacists do not ask patients for a prescription before dispensing antibiotics, which is a concern [10]. These are considered prescription-only medicines as captured in the pharmacy and poisons act [11].

Nyeri County in Kenya is among the top 30% in the country, reporting high rates of COVID-19 transmission. This has resulted in an additional workload on the health system and mental stresses [12] on human resources, rendering it more challenging to adhere to antimicrobial stewardship measures within health facility settings. Studies have reported that the intense hospital use of antibiotics in COVID-19 patients at the early stage of the pandemic [13] may influence bacterial resistance, thereby impacting the capacity to manage infectious diseases in sub–Saharan Africa. The high case fatality rate of 6% against a national target of 2.7% noted in Nyeri Country may partly be due to the high prevalence of non-communicable diseases such as diabetes and hypertension, exacerbated by poor health-seeking behavior. The County also noted delayed reporting to the health facility by suspected community members upon symptoms onset, which may have contributed to increased levels of self-medication and, consequently, the high case fatality rate. Confirmed COVID-19 cases managed under the home-based and isolation care (HBIC) programme are also vulnerable to secondary infections and COVID-19 progression. This may have created an increased need for self-medication using drugs. One of them is Azithromycin, a broad-spectrum macrolide antibiotic that has become a standard treatment for COVID-19 patients even without much evidence [14].

Thus, studies show a rampant use of antibiotics in the treatment of COVID-19, with approximately 75% of COVID-19 patients receiving antibiotics to treat co-infections and secondary infections even though their occurrence remains less than 10% to 15%, respectively [8]. With resources deployed away from antimicrobial stewardship, the indiscriminate use of antibiotics may potentially impact resistance levels if left unchecked with increasing antibiotic resistance and few new antimicrobial agents in the production pipeline [14]. It is thus important to continue surveillance on the population's use of anti-microbials, the source of the drugs, the common drugs used, and their knowledge of the same in the era of COVID-19. Generally, this study seeks to establish the extent of antimicrobial use fueled by self-medication among confirmed COVID-19 cases in Nyeri County, Kenya, most specifically those who might have self-medicated before and after they were confirmed to be COVID-19 positive. The study will address ways of averting this situation amongst community members during prominent public health threats such as the COVID-19 pandemic. It will inform efforts on awareness creation amongst pharmaceutical personnel and consumers to improve understanding of antimicrobial use (AMU) and antimicrobial resistance (AMR). This will ultimately facilitate evidence-based prescription for optimized antimicrobial use.

## Methods

### Study design and location

The study, which considered social distancing requirements, was conducted through phone interviews and consisted of a cross-sectional design targeting respondents sampled from the hospital records of the national list line of COVID-19 positive cases in Nyeri County, Kenya. Notably, the study was undertaken in Nyeri county, given that it was amongst the most affected counties after Nairobi City County in terms of COVID -19 infections. According to the 2019 census of population and housing [15], Nyeri County had a population of 374,288 males and 384,845 females, giving a total population of 759,133. The majority of the people living in Nyeri County are of the Kikuyu Community, primarily engaged in cash and subsistence crop farming, business, or employment in the public and private sectors. It is located in the Kenya Highlands or Mount Kenya region, with agriculture as the main economic activity. The County is also renowned for horticultural farming. The County headquarters is located in Nyeri Town. According to the World Health Organization (WHO) standards, the distribution of health facilities in seven sub-counties is reasonably fair-with most of the sub-county facilities located within a radius of 1–5 km. Health services in Nyeri County are organized across five levels of service delivery that include 118 public health facilities, including "beyond zero" mobile clinics and a hospice for the care of the terminally ill. The County also hosts several private and faith-based health facilities providing a wide range of health services [16].

### Sampling

The complete list of all COVID-19 confirmed cases from August 2020 to July 2021 totaled 2317, which formed the sampling frame for this study. Based on the Fisher sample size calculation formula [17], with finite population correction (FPC), *P* = 0.5 since it is unknown, and a 5% margin of error gave a targeted sample size of 331 respondents. Interviewees from each of the seven sub-counties were determined using probability proportionate to size (PPS) sampling. Systematic random sampling with an interval of five was employed to yield a sample of 280 respondents interviewed for this study.

### Questionnaire and data collection

The first consideration in questionnaire design for this study was the focus of the research identified (during the research problem definition stage and preliminary literature review) to be the extent of antimicrobial use propelled by self-medication during the era of COVID-19. Based on this broad concept, the research team led by the Principal Investigator identified themes within the study topic which then formed the basis for the various modules in the questionnaire. The modules and respective questions in the draft survey instrument were also refined to conform with comparable contemporary studies at the time such as the framework for clarifying self-medication with antibiotics to prevent COVID-19 infection [3] and the CDC listing of COVID-19 symptoms [18]. For validation of the questionnaire, external and technical comments received from the Amref Ethics and Scientific Review Committee (ESRC)—ESRC P1071-2021—were incorporated into the entire research protocol ( including the survey instrument approved). A pilot of the questionnaire and data collection logistics were conducted over two-days. On the first day, under the technical leadership and supervision of the corresponding author, the questionnaire was pre-tested through phone interviews by six research assistants sitting in a central location (yielding 30 filled-out pre-test interviews) in two sub-counties (namely Mukurweini and Tetu) in Nyeri County, sub-counties in which the final interviews would not be conducted but which were similar to the study sub-counties. On the second day, the above research team revised and finalized the research questionnaire based on experiences from day one.

The study used a structured questionnaire (Additional File 1 below) to capture data on socio-demographic characteristics, commonly used antimicrobials, and knowledge and perspective on self-medication. With support from the County health department, trained research assistants telephone-called the sampled respondents to explain the study and seek their consent to be interviewed. A structured questionnaire built on electronic devices using the Open Data Kit (ODK) platform, specifically KoBo Collect, was used for data collection. Completed interviews were securely stored in the institutional server at Amref Health Africa. Six research assistants undertook a two-day face-to-face interviewer training within Nyeri town. The training covered, among other things, phone etiquette and interviewing skills. The research assistants were also trained on the quantitative questionnaire, the consent process, and rapport building.

As part of data quality assurance, each research assistant was allocated, by the data collection supervisor, a target number of interviews to conduct per day. The supervisor and the co-investigator (corresponding author) also checked the questionnaires, as interviews ended, to ensure completeness of filled-out questionnaires.

### Statistical analysis

The data collected were downloaded from the ODK aggregate as an Excel file and exported to STATA version 13 for analysis. Categorical data were summarized using frequencies and percentages. Inferential statistics, including chi-square tests, were used to check for significant differences between categories with variables. Binary logistic regression was used to study the relationship between covariates and the dependent-whether respondents self-medicated or not.

## Results

### Univariate analysis

#### Socio-demographic characteristics of respondents

As shown in Table 1 below, the categories of the respective variables were tested for differences using the Pearson chi-square test. They are all significantly different at the critical level of 5%, except for occupation, which is not. About 34% of the respondents hail from the Nyeri town sub-county, reflecting the urban concentration of the pandemic in the general Kenyan population. Men comprised around 59% of the sample. As far as the age distribution is concerned, age-group 30–39 takes the highest percentage of respondents (32.1%), with proportions decreasing to reach the lowest (12.1%) for those aged 60 years and above. Most of the respondents (73%) are married; those with high school and university education (39.6% and 40%, respectively) representing the educational groups with the most respondents. The high representation of respondents from higher academic categories also highlights the respondents' select nature, which is unique from the rest of the country. Together, those with no income (14.6%) and those earning less than Kshs. 50,000 per month (57.6%) comprise most of the sampled respondents (72.1%). Similarly, a majority of the respondents (87.5%) are registered in the public national health insurance (NHIF) under the universal health coverage (UHC) programme.

**Table 1:** Socio-demographic characteristics of respondents

Sub-county	Freq	Percent	Chi-square
Kieni East	10	3.6	Pr = 0.000
Kieni West	11	3.9	
Mathira East	30	10.7	
Mathira West	5	1.8	
Nyeri Town	94	33.6	
Othaya	28	10.0	
Tetu	12	4.3	
other	90	32.1	
Total	280	100	

Gender	Freq	Percent	
Female	114	40.71	Pr = 0.002
Male	166	59.29	
Total	280	100	

Age group	Freq	Percent	
19–29	55	19.64	Pr = 0.000
30–39	90	32.14	
40–49	50	17.86	
50–59	51	18.21	
60 +	34	12.14	
Total	280	100	

Marital status	Freq	Percent	
Married	200	73.0	Pr = 0.000
Single	74	27.01	
Total	274	100	

Education	Freq	Percent	
Bachelor	112	40.0	Pr = 0.000
Highschool	111	39.7	
None	6	2.1	
Postgraduate	23	8.2	
Primary	28	10.0	
Total	280	100	

Occupation			
Business	41	33.3	Pr = 0.072
Farmers	26	21.1	
Health	34	27.6	
Teachers	22	17.9	
Total	123	100	

Income	Freq	Percent	
50,000–100,000	50	17.86	Pr = 0.000
< 50,000	161	57.5	
> 100,000	28	10	
No income	41	14.64	
Total	280	100	

NHIF-UHC*	Freq	Percent	
No	35	12.5	Pr = 0.000
Yes	245	87.5	
Total	280	100	

Private insurance	Freq	Percent	
No	173	61.8	Pr = 0.000
Yes	107	38.2	
Total	280	100	

*National Hospital Insurance Fund (NHIF)-Universal Health Coverage (UHC) registration

On the contrary, 38.2% are covered under private health insurance. It is appropriate to mention that Nyeri County was among the four out of the 47 counties in the country that piloted the UHC programme, giving it a head-start in UHC enrolment. The select nature of the respondents (a substantial proportion being those with higher educational levels and salaried incomes) may also explain the relatively higher ownership of private medical insurance cover presumably provided by the employer.

#### Distribution of patients who developed COVID-19 symptoms

**Fig. 1:**
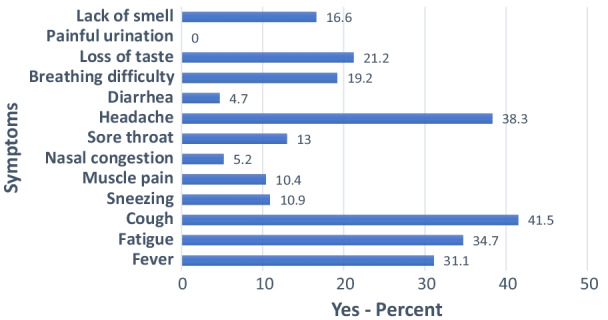
Percentage distribution of respondents indicating COVID-19 symptoms

Out of the 280 respondents interviewed and all of whom were PCR test confirmed COVID-19 cases, 193 (68.9%) indicated that they developed some COVID-19 symptoms. Figure 1 beow shows the percentage distribution of respondents who self-reported “yes” to developing the listed COVID-19 symptoms. Of the well-documented COVID-19 symptoms [18] captured in the questionnaire, cough (41.5%), headache (38.3%), and fatigue (34.7%) were the leading symptoms reported by the respondents. As expected, undocumented symptoms such as painful urination were not mentioned by any respondents.

### Self-medication with commonly-used microbial agents

Coming to the core of the study, respondents were asked if they self-medicated with antibiotics during this time of COVD-19. As shown in Table 2 below, 66 out of the 280 respondents (23.6%) indicated that they did. Among the 66 who reported self-medication, asked further whether they self-medicated before or after their respective COVID test, the majority (60.6%) indicated that they did so before the test. Almost half of the self-medicated respondents (48.5%) did so once, with the complement (51,2%) indicating that they self-medicated more than once. The dynamics around self-medication captured in the survey are also interesting. Seventy-five percent (75%) of those who obtained medication did so from a pharmacy, implying the need for more stringent pharmaceutical outlet regulation. 63.3% of those who self-medicated procured antibiotics (Amoxycillin and Azithromycin), with 21.7% indicating that they could not remember the name of the drug they obtained from the dispensing outlet. Respondents indicated that their choice of the drugs is mainly based on their own experience (26.7%), recommendations of community pharmacists (25%), and opinions of friends as well as family members (18.3%). The need for education on instructions for the use of antimicrobials is evident from the survey results. Up to 66.6% of the respondents who obtained antimicrobials from a dispensing outlet either never checked (43.3%) the package or leaflet instructions or did so only sometimes (23.3%).

**Table 2:** Medication with commonly-used antimicrobial drugs

Treated self	Freq	Percent
No	214	76.43
Yes	66	23.57
Total	280	100

When self-treated	Freq	Percent
After test	26	39.39
Before test	40	60.61
Total	66	100

Times	Freq	Percent
1	32	48.48
> 1	34	51.52
Total	66	100

Where obtained medicine	Freq	Percent
Pharmacy	45	75.0
Other *	15	25.0
Total	60	100

Which medicine	Freq	Percent
Amoxycilline	14	23.3
Azithromycin	24	40.0
Can’t remember	13	21.7
Others**	9	15.0
Total	60	100

Selection based on	Freq	Percent
Own experiences	16	26.7
Opinion of friends/family	11	18.3
Previous doctor's prescription	5	8.3
Recommendation by community pharmacists	15	25.0
other	13	21.7
Total	60	100

Checked instructions	Freq	Percent
I was not given	1	1.7
Never	26	43.3
Yes, sometimes	14	23.3
Yes, always	19	31.7
Total	60	100

*Includes Government and private hospitals

**Comprises Amoxyclav, Clarithromycin, and Tetracyclines

### Knowledge, attitude, and practices on self-medication

From the responses to the question on awareness about regulations related to antimicrobial self-regulations (Table 3), it is apparent that awareness of self-medication is about equally spread. Thus, only 50.4% of the respondents indicated that they knew antimicrobial use regulations. In contrast, among the smaller number (60) of respondents who had obtained antimicrobial medication, virtually all (55 out of 60 or 91.7%) respondents knew the dosage when taking their antibiotics. This knowledge was gained through consulting a doctor (38.2%), a pharmacist (27.3%), or the package insert (20%). Forty-five percent (27/60) of these were familiar with the dosage since they usually use similar drugs whenever ill. Among the whole sample of respondents, a smaller proportion (65%) know the duration of taking antimicrobial medicines. Some (23.6%) would recommend the treatment they took to someone else in their community. Virtually all (234/254 or 92%) of the respondents think that self-medication with antimicrobials for self-health care is unacceptable and is associated with health risks. An even higher proportion (96.8%) believe that one should complete medication as a best practice as advised by the medical practitioner. During the interviews, several options were floated that would address the problem of ant-microbial self-use among the population. 68.1% of the respondents advocated for the need to create awareness of antimicrobial use, as highlighted in (Table 3 below).

**Table 3:** Distribution of respondents on knowledge, attitude, and practice (KAP) aspects of antimicrobial self-medication

Aware of regulations towards antibiotic self-medication	Freq	Percent
No	126	49.6
Yes	128	50.4
Total	254	100

Knew dosage at time when taking antibiotics?	Freq	Percent
No	5	8.3
Yes	55	91.7
Total	60	100

How knew dosage?	Freq	Percent
By checking the package insert	11	20.0
By consulting a health care provider	21	38.2
By consulting a dispensing personnel	15	27.3
Other*	8	14.5
Total	55	100

Know duration of taking antimicrobial medicines?	Freq	Percent
No	89	35.0
Yes	165	65.0
Total	254	100

Would recommend treatment taken to someone in community?	Freq	Percent
No	194	76.4
Yes	60	23.6
Total	254	100

Normally use similar drugs whenever ill?	Freq	Percent
No	33	55.0
Yes	27	45.0
Total	60	100

What do you think of self-medication with antibiotics for self-health care?	Freq	Percent
Acceptable practice	13	5.1
Good practice	7	2.8
Not acceptable	234	92.1
Total	254	100.0

Do you think antibiotic self-medication is associated with risks?	Freq	Percent
No	20	7.9
Yes	234	92.1
Total	254	100

What are the best practices while taking antimicrobial medicines?	Freq	Percent
You finish the medication as directed by a health practitioner	246	96.8
You discard the remaining left-over medications	2	0.8
You leave medication immediately you feel better	1	0.4
You save medication to take next time you become ill	1	0.4
Other	4	1.6
Total	254	100

What to be done-create awareness	Freq	Percent
No	81	31.9
Yes	173	68.1
Total	254	100

What to be done-implement laws on self-medication	Freq	Percent
No	206	81.1
Yes	48	18.9
Total	254	100

What to be done-advocate for behavioral change	Freq	Percent
No	241	94.9
Yes	13	5.12
Total	254	100

^*^Includes consulting family member/friend; from previous experience

### Multivariate analysis-logistic regression results

As shown in Table 4 below, four factors, three under the knowledge, attitude, and practice (KAP) group and one under the socio-demographic cluster, were significantly related to self-medication. These are developing COVID symptoms, awareness of regulations on antimicrobial self-medication, the need to create awareness about the risks of self-medication, and age group. Of these four, respondents who developed COVID symptoms were surprisingly 9.5 times more likely to self-medicate than those who reported not having developed symptoms, the significance being at the 1% level. Similarly, being aware of regulations on self-medication with antibiotics and creating awareness of self-medication risks have odds ratios of 3.1 and 4.6, both corresponding significance of 5%. Being older than 19–29 years is generally significantly related to reduced self-medication with antibiotics. The odds ratios are 0.04, 0.05, and 0.007 for age groups 30–39, 50–59, and 60 + relative to the reference category of 19–29 years. The results are significant at the 1% (age groups 30–34 and 60 +) and 5% (age category 50–59) levels.

**Table 4:** Logistic regression of self-treatment on covariates

Dep. = Self-treated 0 = No; 1 = Yes	Coef	Odds ratio	SE	*P*-value	[95% Conf	Interval]	Sig
*Sub-county* Ref. = Nyeri town							
Other areas	− 0.093	0.912	0.64	0.885	− 1.346	1.161	
*Gender* Ref = Female							
Male	0.199	1.220	0.546	0.716	− 0.871	1.269	
*Age group* Ref. = 19–29 years							
30–39	− 3.218	0.040	1.029	0.002	− 5.235	− 1.202	**
40–49	− 1.344	0.261	1.065	0.207	− 3.431	0.743	
50–59	− 2.937	0.053	1.202	0.015	− 5.293	− 0.581	*
60 +	− 4.909	0.007	1.68	0.003	− 8.2	− 1.617	**
*Marital status* Ref. = Married							
Single	− 0.509	0.601	0.784	0.516	− 2.046	1.027	
*Education* Ref. = High school and less							
Degree or postgraduate	− 0.769	0.463	0.719	0.284	− 2.178	0.639	
*Occupation* Ref. = Business							
Farmers	0.545	1.724	0.929	0.558	− 1.276	2.365	
Health	− 0.662	0.516	0.799	0.407	− 2.229	0.904	
Teachers	− 0.748	0.473	0.94	0.426	− 2.591	1.095	
*Income* Ref. = 50–100 k							
< 50,000	− 0.139	0.870	0.805	0.863	− 1.717	1.439	
> 100,000	0.100	1.105	1.072	0.926	− 2.002	2.202	
No income	− 1.333	0.264	1.466	0.363	− 4.207	1.541	
*Private insurance* Ref. = No							
Yes	0.719	2.053	0.668	0.282	− 0.59	2.029	
*Covid symptoms* Ref. = No							
Yes	2.247	9.461	0.815	0.006	0.65	3.844	**
*Aware of regulations* Ref. = No							
Yes	1.117	3.056	0.558	0.045	0.024	2.21	*
*Associated with risk* Ref. = No							
Yes	0.169	1.184	1.183	0.887	− 2.149	2.487	
*What to be done: create awareness* Ref. = No							
Yes	1.533	4.632	0.616	0.013	0.326	2.74	*
*What to be done: Implement laws* Ref. = No							
Yes	0.62	1.858	0.64	0.333	− 0.635	1.874	
Constant	− 1.597	0.203	2.018	0.429	− 5.552	2.357	

*P < 0.05, **P < 0.01

Although not statistically significant, several other variables are associated with reduced self-medication odds. These include other areas relative to Nyeri town sub-county, single marital status compared to being married, respondents with a degree or higher educational attainment compared to those who have a high school or lower education, those working in the health and teaching profession compared to those in business, and those with no income or income less than Kshs. 50,000 relative to those with 5000–100,000. On the other hand, a number of variables are related to increased odds of self-medication. These are being male, owning private insurance relative not owning such insurance, associating self-medication with risks, and suggesting that laws on self-medication should be enacted. 

## Discussion

- Significance of the study
- The most significant and influential finding in this study, on 280 respondents confirmed with COVID-19, is that those who reported having developed COVID-19 symptoms are more likely to self-medicate with antibiotics. Pointedly, results of the frequency distribution (Table 2) also show that out of the 66 respondents who self-medicated, 60.6% did so before taking the PCR test and knowing the results. The world is experiencing an increased risk of infectious disease outbreaks due to, among other factors, increased urbanization, spillover from animals to humans, increased travel and trade, weak public health infrastructure, and antimicrobial resistance. It is paramount that public health actors address the increased antimicrobial misuse during health emergencies by putting in place necessary measures such as policies and community and health worker sensitization on the risk of AMR to reduce the double threat to health security.

### Interpretation of significant findings

This causal linkage between symptoms and self-medication has also been reported in similar studies on self-medication during the COVID-19 response. Similar to this study in Kenya, common symptoms of self-medication included fever, throat pain, and dry cough in one study in Bangladesh [21]. In another study in Pakistan, fever, muscle pain, fatigue, sore throat, and cough were the symptoms indicated for resorting to self-medication [22]. This finding, completed by literature review, calls for the revision of health policies to include the need for increased awareness of the risks of self-medication, especially during health emergencies. The 23% self-medication rate established in this study among the confirmed cases in Nyeri County can be contrasted to a recent finding by [23], where an increase in self-medication from 36.2% before to 60.2% during the pandemic was noted among health workers in Kenya. Yet, [24] in their systematic review of eight international studies on self-medication, report a wide range of self-medication prevalence in the general population, ranging from less than 4% to 88.3%. These studies buttress the ubiquitous observation of heightened self-medication and its contribution to AMR during health emergencies such as the recent COVID-19 pandemic. Further, they suggest that the 23% prevalence of self-medication among those diagnosed with COVID-19 in this study might even be a lower limit and could be higher.

Research results on self-medication among the general population in Nigeria [25] show that 41% of respondents self-medicated to prevent and treat COVID-19. The contributing factors in the Nigerian study are particularly worth sharing, and they include fears of stigmatization, discrimination, quarantine, infection, or contact with a person suspected to be infected with COVID-19. Primary reasons for self-medication comprised the emergency nature of the illness, delay in access to hospital services, long distance to the health facility, and closeness of the pharmacy. Similar results emerge in Kenya [26] in a study among pharmacy customers and pharmacy workers: the primary motivator for self-medication is fear of visiting a medical facility and the perceived experience once one reaches there. The reasons for self-medication in our study may well be similar to those found above in the two studies in Nigeria and Kenya.

Perplexing, though, were the positive and significant relationships noted between two independent KAP variables and the dependent variable (self-medication). These covariates are respondents who indicated that they are aware of regulations on antibiotic self-medication and those who recommended the creation of more education on the risks of self-medication. Common sense would dictate that those aware of regulations on the use of antimicrobials and those advocating for more education would be wary and less likely to self-medicate. On the contrary, the study results suggested the opposite, with those knowledgeable of the regulations and those recommending more education more likely to self-medicate, and the critical question is why? In a departure from most self-medication studies that dwell on KAP, psychological distress and perceived health risks in explaining antibiotic self-medication have been noted in studies such as [3] in Australia. The study finds that psychological anguish-most notably emotional reactions of "panicked" and "scared"—induced by the COVID-19 pandemic is positively and significantly linked with self-medication. Thus, even though persons who exhibit COVID-19 symptoms are aware of self-medication regulations, the immediate behavioral and emotional response in this distressing environment would be enough to influence their self-medication. The reasons for self-medication are varied and partially fall under supply-side issues such as perceptions of delays in receiving treatment, lack of medication at the health facility, the lower cost of medicines in the drug outlets, and lack of observance of regulations in local drug outlets. Demand-side factors such as fears-dreading that one might be put under quarantine or self-isolation and discriminated against by others-are other reasons for self-medication. Thirdly, peer pressure and social media influence also play a role in self-medication at the interpersonal level.

(iii) **Implications**

There is considerable literature [27–29] affirming the continuation or future recurrence of health emergencies such as COVD-19. The results of this study expose programming, policy, and research gaps that need to be addressed for better preparedness, prevention, and response (PPR) to health emergencies.

(iv) **Study limitations**

The strengths and limitations of this study should be viewed in the context of the heavily constrained environment of COVID-19 in which it was conducted. On the downside, getting the respondents (many of who may have been in poor health due to COVID-19) to answer all the questions using the phone interview was not 100% successful, with the result being that some questions had missing values. Nevertheless, this shortcoming is largely overcome by the encouraging results of the descriptive statistics and causal inferences outlined above.

## Conclusions

In the sample of 280 confirmed COVID-19 cases, 23.4% reported self-medication with antimicrobials, and among these, 60.6% did so before the test, and 51.5% self-medicated more than once. Common antimicrobials self-medicated were Azithromycin (40.0%) and Amoxycilline (23.3%); those who could not remember the drug they received also were considerable (21.7%). 193 (68.9%) of all respondents indicated developing symptoms-mainly cough (41.5%), headache (38.3%), and fatigue (34.7%). Only 50.4% of the respondents (128/254) know the regulations towards self-medication with antimicrobials. Increasing age was significantly related to reduced self-medication. On the other hand, developing COVID-19 symptoms, awareness of COVID-19 regulations, and perception of a need to create more awareness of self-medication were related to a higher likelihood of self-medication.

Further research needs to be conducted on the self-medication and AMR nexus, especially during health emergencies. Secondly, it will be necessary to integrate the self-medication dynamics unraveled in this study at the policy level into AMR policies and guidelines. Lastly, for programme implementation, self-medication messaging should be timely. It should also include awareness creation on the impact of self-medication on AMR, including the psychological factors in clients and service providers that come into play during such health emergencies.

## Supplementary Information

**Additional file 1**: Final Questionnaire III.

## Data Availability

The questionnaire is attached herewith as Additional file 1. Data collected and used for analysis, as well as the STATA code are available through the corresponding author upon request.

## Abbreviations

AMR: Antimicrobial resistance
AMU: Antimicrobial use
CHMT: County Health Management Team
COVID-19: Coronavirus disease-19
FPC: Finite population correction
HBIC: Home-Based and Isolation Care
KAP: Knowledge, attitude and practice
Kshs: Kenya shillings
ODK: Open data kit
PPS: Probability Proportional to Size
PPR: Preparedness, Prevention, and Response
SMA: Self Medication with Antibiotics
STATA: Statistics and data software
UHC: Universal health coverage
