# Supplementary material for: A cross-sectional study of antimicrobial use among self-medicating COVID-19 cases in Nyeri County, Kenya
Source: Antimicrob Resist Infect Control. 2022 Aug 30;11:111. doi: 10.1186/s13756-022-01150-7 (PMC9427085; doi:10.1186/s13756-022-01150-7)
Supplement: Supplementary file 1 — Additional file 1: Final Questionnaire III. [file 13756_2022_1150_MOESM1_ESM.docx]

## INFORMED CONSENT FORM

**Study Title:** **Investigating the extent of Anti-Microbial Use amongst Self-Medicating COVID-19 cases in Nyeri County.**

**Dear Participant**

My name is **_______________**working as a research assistant for Amref Health Africa in collaboration with County Government of Nyeri. We would like to collect information regarding self-medication on antibiotics.

**Broad objective of the study**

This study seeks to establish the extent of anti-microbial use fueled by self-medication among confirmed COVID-19 cases in Nyeri County, Kenya with the aim of suggesting evidence-based intervention to reduce the practice.

**Potential Risks**

We do not anticipate any risks or discomforts to you during this study. We will make every effort to protect your privacy and confidentiality while you are participating in the study. The interview will take place in private.

**Potential Benefits**

With these findings, we shall have evidence-based data to advise relevant health authorities and policy makers in coming up with solutions self-medication and use of antibiotics .

**Confidentiality**

Your names will not appear in any of the reports from this study. No identity of any specific individual will be disclosed in any public reports or publications.

**Participation**

Participation is voluntary hence should you agree to participate in this study, kindly sign the section at the end of this form.

_______________________________________ ________________________________

**Signature/Thumbprint of Study Participant** **Date**

_______________________________ _________________________________

**Signature of Person Obtaining Consent** **Date**

For any questions or concerns about this study or in the event of a study related injury, kindly contact the Principal Investigator Jackie Kiarie on +254 20 699 4000, of Amref Health Africa Kenya. Email: [jackline.Kiarie@amref.org](mailto:jackline.Kiarie@amref.org)

**Questionnaire for self-medication with anti-microbials**

| **Number** | **Question** | **Response** | **Skip** |
| --- | --- | --- | --- |
| **Demographic Characteristics** | | | |
|  | Sub County of Origin | 1. Nyeri Central 2. Mathira East 3. Othaya 4. Kieni East |  |
|  | Gender | 1. Male 2. Female |  |
|  | Age (Years) | _____________ |  |
|  | Marital status | 1. Single 2. Married 3. Divorced 4. Widowed 5. Separated |  |
|  | Education level | 1. None 2. Primary 3. High school 4. Bachelor 5. Post graduate |  |
|  | Monthly income | 1. < Ksh 50,000 2. Ksh 50,000-100,000 3. >Ksh 100,000 4. No income |  |
|  | What is your occupation | 1. Students 2. Civil service 3. Non civil service 4. Self-employed/business 5. Retired 6. Unemployed 7. Other Specify__________ |  |
|  | Are you enrolled in NHIF/UHC? | 1. Yes 2. No |  |
|  | Do you have a private health insurance? | 1. Yes 2. No |  |
| **Commonly used anti-microbial agents** | | | |
|  | Did you develop any COVID_19 symptom(s) | 1. Yes 2. No | **If No End the Interview** |
|  | What were the symptoms?  *Tick all that apply* | 1. Fever 2. Fatigue 3. Cough 4. Sneezing 5. Muscle pain 6. Nasal congestion 7. Sore throat 8. Headache 9. Diarrhea 10. Breathing difficulty 11. Loss of taste 12. Painful urination 13. Lack of smell 14. Other (Specify)_______________ |  |
|  | Did you treat yourself (self-medication) with antibiotics during this time of COVID 19? | 1. Yes 2. No |  |
|  | Did you buy the recommended dosage? | 1. Yes 2. No 3. I don’t Know |  |
|  | When did you self-medicate? | 1. Before COVID-19 2. After COVID-19 |  |
|  | How many times did you treat yourself with antibiotics? | _________________________ |  |
|  | Did you complete the treatment? | 1. Yes 2. No |  |
|  | If positive, what was the reason for self-medication? *(Multiple response)* | 1. Fear of stigma or discrimination 2. Fear of being quarantine or self-isolated 3. Delay in receiving treatment at health facilities 4. Influence of friends to use self-medication to treat COVID 19 5. Influence from social media messages 6. Influence from television/radio and newspaper 7. No drugs and treatment for COVID 19 in the health facilities 8. Less expensive   Others (Specify)_____ |  |
|  | Did the medication relieve the symptom you had? | 1. Yes 2. No | 1. Yes   No |
|  | To what extent did they relieve the symptoms? | 1. To a great extent 2. Somewhat 3. Very little change |  |
|  | Where did you get the medicine? | 1. Pharmacy 2. Faith Hospital 3. Private hospital 4. Government hospital 5. Left over from previous prescription 6. Online shopping/E-pharmacies 7. Other specify______________ |  |
|  | If hospital, which one? | PGH  Karatina District Hospital  Others (Specify)_____ |  |
|  | Which medicines were you given at the hospital?  *Only for those who visited health facility* | 1. Azithromycin 2. Hydroxychloroquine 3. Amoxyclav 4. Clarithromycin 5. Ciprofloxacin 6. Cefuroxime 7. Cefixime 8. Tetracyclines 9. Don’t Know 10. Can’t remember 11. Others (Specify)_________________ |  |
|  | What was your selection of antibiotics was based on? (*Tick all that apply)* | 1. Recommendation by community pharmacists 2. Opinion of family members 3. Opinion of friends 4. My own experiences 5. Previous doctor’s prescription 6. The advertisement |  |
|  | What did you consider when selecting antibiotics ? (Tick all that apply) | 1. Type of antibiotics 2. Brand of antibiotics 3. Price of antibiotics 4. Indications for use 5. Adverse reactions 6. Others (specify)______________ |  |
|  | Did you ever check the instructions come with the package insert of antibiotics for self-medication treatment? | 1. Yes, always 2. Yes sometimes 3. Never 4. I was not given | **If Never Skip to Q 26** |
|  | How much did you understand the instructions? | 1. Fully understood 2. Partly understood 3. Did not understand at all |  |
| **Knowledge and attitude on Self Medication** | | | |
|  | Are you aware of any regulation towards use of antibiotic self-medication? | 1. Yes 2. No |  |
|  | Did you know the dosage at the time you were taking the antibiotics ? | 1. Yes 2. No |  |
|  | How did know you the dosage? | 1. By checking the package insert 2. By consulting a doctor 3. By consulting a pharmacist 4. By consulting a family member/friend 5. From the media (Newspaper/magazine/books.TV/Radio 6. From the internet 7. From my previous experience 8. By guessing the dosage, myself 9. Other (Specify)________________ |  |
|  | Do you know the duration of taking anti-microbial medicines? | 1. Yes 2. No |  |
|  | Would you recommend the same treatment you took to someone in your community? | 1. Yes 2. No |  |
|  | Do you normally use similar drugs whenever you are ill? | 1. Yes 2. No |  |
|  | What do you think about self-medication with antibiotics for self-health care? | 1. Good practice 2. Acceptable practice 3. Not acceptable practice |  |
|  | Do you think antibiotic self-medication is associated with risks? | 1. Yes 2. No | **If No Skip to Q 35** |
|  | If Yes, what are some of the risks? | 1. Wastage of money 2. Resistance 3. Side effects 4. Masking symptoms of underlying disease 5. Other (Specify)_________________ |  |
|  | What are the best practices while taking anti-microbial medicines? (*Multiple response*) | 1. You finish the medication as directed by a health practitioner 2. You save medication to take next time you become ill 3. You discard the remaining left-over medications 4. You leave medication immediately you feel better 5. You donate the left-over medication to a friend who becomes ill 6. Other (Specify)________________ |  |
|  | What do you think should be done to solve the anti-microbial self-medication practice among the population? | 1. Create awareness on the risks of self-medication 2. Implement the laws on self-medication 3. Advocate for behavioral change programs on self-medication 4. Improving the understanding of label and leaflet accompanying medication 5. Others……... |  |

********The End********
